# Increasing water availability and facilitation weaken biodiversity–biomass relationships in shrublands

**DOI:** 10.1002/ecy.2624

**Published:** 2019-02-25

**Authors:** Yanpei Guo, Christian Schöb, Wenhong Ma, Anwar Mohammat, Hongyan Liu, Shunli Yu, Youxu Jiang, Bernhard Schmid, Zhiyao Tang

**Affiliations:** ^1^ Institute of Ecology College of Urban and Environmental Sciences and Key Laboratory for Earth Surface Processes Peking University Beijing China; ^2^ Department of Evolutionary Biology and Environmental Studies University of Zurich Zurich Switzerland; ^3^ Department of Environmental Systems Science Swiss Federal Institute of Technology ETH Zurich Zurich Switzerland; ^4^ School of Life Sciences Inner Mongolia University Hohhot China; ^5^ Xinjiang Institute of Ecology and Geography Chinese Academy of Sciences Urumqi China; ^6^ State Key Laboratory of Vegetation and Environmental Changes Institute of Botany Chinese Academy of Sciences Beijing China; ^7^ Key Laboratory of Forest Ecology and Environment State Forestry Administration, Research Institute of Forest Ecology Environment and Protection Chinese Academy of Forestry Beijing China; ^8^ Department of Geography University of Zurich Zurich Switzerland

**Keywords:** biodiversity–ecosystem functioning relationships, biomass, competition, facilitation, shrub density, shrublands, water availability

## Abstract

Positive biodiversity–ecosystem‐functioning (BEF) relationships are commonly found in experimental and observational studies, but how they vary in different environmental contexts and under the influence of coexisting life forms is still controversial. Investigating these variations is important for making predictions regarding the dynamics of plant communities and carbon pools under global change. We conducted this study across 433 shrubland sites in northern China. We fitted structural equation models (SEMs) to analyze the variation in the species‐richness–biomass relationships of shrubs and herbs along a wetness gradient and general liner models (GLMs) to analyze how shrub or herb biomass affected the species‐richness–biomass relationship of the other life form. We found that the positive species‐richness–biomass relationships for both shrubs and herbs became weaker or even negative with higher water availability, likely indicating stronger interspecific competition within life forms under more benign conditions. After accounting for variation in environmental contexts using residual regression, we found that the benign effect of greater facilitation by a larger shrub biomass reduced the positive species‐richness–biomass relationships of herbs, causing them to become nonsignificant. Different levels of herb biomass, however, did not change the species‐richness–biomass relationship of shrubs, possibly because greater herb biomass did not alter the stress level for shrubs. We conclude that biodiversity in the studied plant communities is particularly important for plant biomass production under arid conditions and that it might be possible to use shrubs as nurse plants to facilitate understory herb establishment in ecological restoration.

## Introduction

The biodiversity–ecosystem‐functioning (BEF) relationship has been a major focus of ecological research in recent decades (Naeem et al. 1994, Tilman et al. 1996, Cardinale et al. 2006, Erskine et al. 2006, Beaugrand et al. 2010, Vaughn 2010, Chen et al. 2018). Using annual net primary productivity (ANPP) as the ecosystem function of interest, a large number of studies conducted in herbaceous communities have found positive BEF relationships (Fargione et al. 2007, Isbell et al. 2009, Marquard et al. 2009, Ma et al. 2010, Craven et al. 2016). Similarly, positive correlations between biodiversity and biomass have been found in forests in different climatic regions (Wardle et al. 2012, Cavanaugh et al. 2014, Castro‐Izaguirre et al. 2016, Liang et al. 2016, Adair et al. 2018, Liu et al. 2018).

Drivers of global change, such as increasing temperature and nitrogen deposition, not only directly affect biodiversity but may also modify BEF relationships (Ammer 2018, Paquette et al. 2018). Therefore, understanding the variation in BEF relationships along environmental gradients is of major importance and could provide useful information for the management of natural ecosystems in terms of biodiversity and carbon stock conservation. Previous studies have shown divergent results in terms of the variation in BEF relationships in different environments. Although a series of nutrient addition experiments in herbaceous communities (He et al. 2002, Fridley 2003, Wacker et al. 2009, Yin et al. 2017) found that the positive effects of biodiversity on productivity become stronger under more favorable conditions, some observational studies in natural forests suggested the opposite, indicating that positive BEF relationships become weaker in more favorable habitats (Paquette and Messier 2011, Potter and Woodall 2014, Wu et al. 2015). The latter is usually explained by the competitive exclusion of subordinate species due to the increased growth of dominant species, especially when there is niche overlap and functional redundancy between subordinates and dominants (Warren et al. 2009, Paquette and Messier 2011, Wu et al. 2015). This change is also compatible with the so‐called stress‐gradient hypothesis, which suggests that competition is less intense and that positive interactions are more important in stressful environments than in benign habitats (Bertness and Callaway 1994, Choler et al. 2001, Callaway et al. 2002); thus, less positive BEF relationships in association with decreasing environmental stress are expected (He et al. 2013).

Past studies on BEF relationships have mainly focused on a single life form (Fargione et al. 2007, Weigelt et al. 2010, Ruiz‐Jaen and Potvin 2011, Ruiz‐Benito et al. 2014). However, in forests and shrublands, communities are composed of species of different life forms, with various interactions among them. For instance, shrubs can compete against herbs for resources, but under certain environmental conditions, they can also facilitate herbs by improving soil water and nutrient conditions (Miller and Gorchov 2004, Noumi et al. 2016). While facilitative interactions improve the growth conditions of species, competitive interactions do the opposite (Schenk 2006, Bronstein 2009, McIntire and Fajardo 2014, Aschehoug et al. 2016). Facilitative or competitive effects of one life form on another may thus weaken or strengthen positive BEF relationships, respectively, because they alter the stress level for the responding life form in opposite directions. Moreover, facilitative and competitive effects can depend on the biomass of the interacting groups, which has been demonstrated in alpine cushion plant systems, where larger cushions have stronger facilitative effects, while the greater cover of species benefitting from the facilitation inhibits the cushions more severely (Schöb et al. 2013a, 2014).

In the present study, we use shrublands in northern China as an example to test two hypotheses derived from the information presented above. First, we predict a positive BEF relationship between species richness and biomass for shrubs and herbs under arid, i.e., more stressful, conditions, which will be weakened or even become negative under wetter, i.e., more benign, conditions. Second, assuming that shrubs facilitate herbs, while herbs compete against shrubs (Facelli and Pickett 1991, Kunstler et al. 2006, Gómez‐Aparicio 2009, Cuesta et al. 2010), we hypothesize that an increase in shrub biomass might weaken the positive species‐richness–biomass relationship for herbs because it creates more benign conditions for herbs, while increasing herb biomass might strengthen the positive species‐richness–biomass relationship for shrubs because it creates more stressful conditions for shrubs.

The investigated shrublands cover a wetness/aridity gradient across northern China, from the humid monsoon region in the east to the dry desert region in the west. Because water availability is the key limitation regarding the distribution of vegetation in northern China (Bai et al. 2008, Ma et al. 2010), we used climatic wetness as the environmental stress factor to test our first hypothesis. Shrubland, as a vegetation type that is less often studied than grassland and forest, has an obvious advantage in terms of addressing our second hypothesis. Shrubland consists of both herbs and shrubs, which allows us to identify modifications to the species‐richness–biomass relationship exhibited by one group resulting from the amount of biomass presented by the other group.

## Materials and Methods

### Site investigation and plant measurements

**Figure 1:**
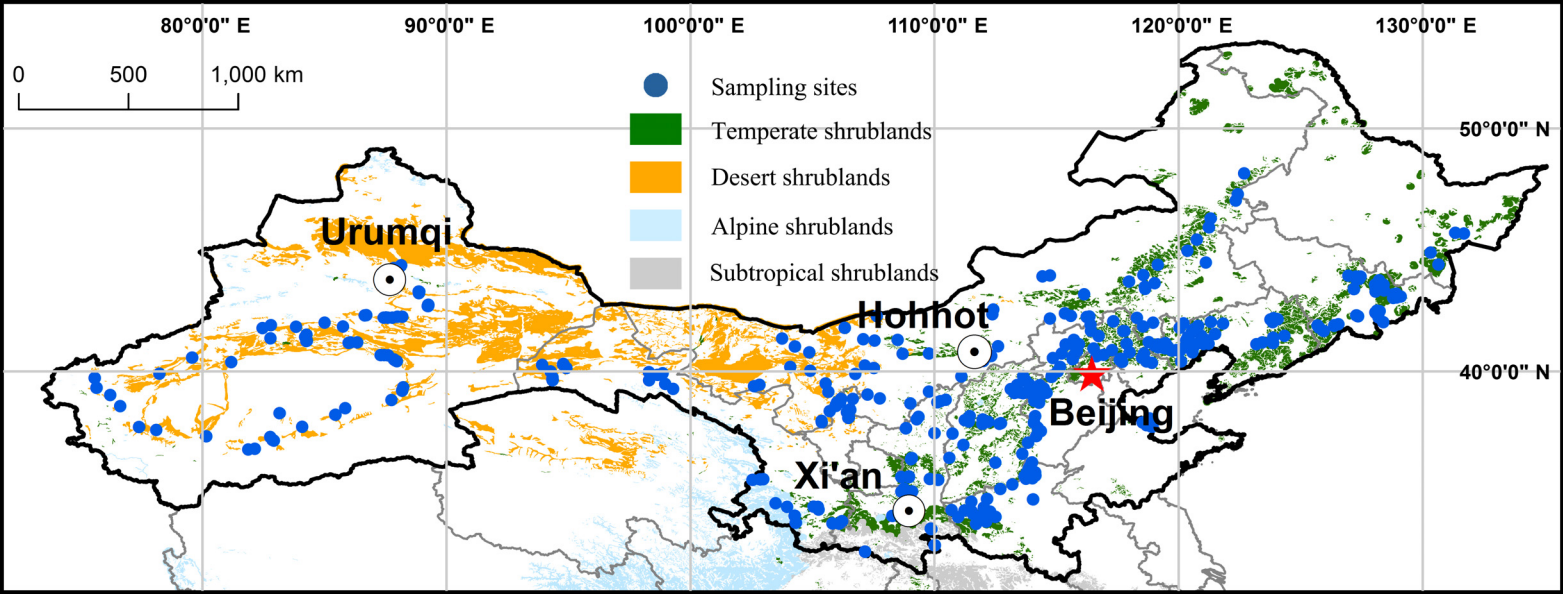
Site locations in northern China based on a shrubland distribution map (Editorial Committee of Vegetation Map of China 2007).

We conducted a survey of 433 natural shrubland sites across northern China, with a geographical range of 32.6–45.9° N latitude and 75.6–131.7° E longitude (Fig. 1). Based on the shrubland distribution map in the *Vegetation Map of P. R. China (1:1,000,000)* (Editorial committee of Vegetation Map of China 2007), we divided our study region into 15′ × 10′ grid cells and identified the cells in which shrublands covered at least 30% of the area. For each province in northern China, 3% of the qualified cells were randomly chosen, and the locations of the investigation sites in these cells were determined according to historical information about the local vegetation. The field investigation was conducted between July and September of 2011, 2012 and 2013. At each site, we investigated the shrub composition in three plots of 5 × 5 m (10 × 10 m in desert shrublands due to the sparse distribution of shrubs) and the herb composition in four 1 × 1 m subplots at the four corners of each plot. The distances between plots within one site were 5–50 m. We used species richness as the measure of the biodiversity of shrubs and herbs. The species richness at one site was calculated as the total number of plant species that we found in three plots (for shrubs) or 12 subplots (for herbs). We used a species**–**area relationship for desert shrublands (*S* = *c* × *A* ^0.28^, where *S* represents species richness and *A* represents area) to correct the shrub richness for the larger plot size in desert shrublands, which was established based on 12 nested plots in the same region (Xinjiang Autonomous Region; Qiao et al. 2011).

We measured the basal area, height, and crown diameter of each shrub individual in each plot and used these morphological parameters to estimate the biomass of each shrub individual according to the biomass models in the *Manual of Biomass Models for Common Shrubs in China* (Xie et al. 2018). This manual contains biomass models for different shrub species in different regions, and the models that we used were built based on the shrub samples from our shrubland investigation. The biomass calculations included two steps. First, the aboveground biomass was calculated according to corresponding biomass models with morphological parameters, i.e., aboveground biomass was modeled as a function of the morphological parameters. Second, the belowground biomass was calculated according to allometric biomass models with aboveground biomass, i.e., belowground biomass was a function of the aboveground biomass. The sum of the above‐ and belowground biomasses of all individuals at one site was considered as the total shrub biomass at that site. For some dense shrubs for which it was difficult to identify individuals, we harvested the plants, weighed the total fresh mass, and took samples of the roots, stems and leaves of each species. The samples were then taken to the laboratory, oven dried at 65°C, and then weighed to obtain the dry mass. We also harvested all herbs in all 1 × 1 m subplots and repeated the same procedure used for the harvested shrubs. The dry mass of the roots, stems, and leaves of dense shrubs and all herbs was calculated as follows:

(1) $$\begin{array}{cc} \text{Total dry mass}=\; & \text{Sample dry mass}/\text{Sample fresh}\\ & \text{mass}\times \text{Total fresh mass}. \end{array}$$

The sum of the total dry masses of the three organs was considered the total biomass of the dense shrubs or herbs at one site. We corrected the biomass for different plot sizes by calculating the biomass per square meter at each site.

### Soil sampling and measurements

Three 1‐m‐deep pits (or <1 m in depth when we reached bedrock) were excavated along the diagonal of each plot. We took soil samples at depths of 0–10, 10–20, and 20–30 cm and mixed them together to obtain three samples taken at the same depth from a single plot. All samples were air dried and the roots were removed. Soil total nitrogen (STN) was measured using an elemental analyzer (2400 II CHNS; Perkin‐Elmer, Boston, Massachusetts, USA), and soil total phosphorus (STP) was measured using the molybdate/ascorbic acid method after H_2_SO_4_‐H_2_O_2_ digestion (Jones 2001). STN and STP in the top 30 cm were used as indicators of the soil nutrient conditions. In the absence of available N and P data, we considered STN and STP as useful surrogates for the soil nutrient conditions for the following reasons. First, studies have shown that STN is positively correlated with the N mineralization rate (Bertiller et al. 2006), and both values are used as indicators of soil fertility at large spatial scales (Wang et al. 2009, Liu et al. 2013), so they can represent the soil nutrient conditions to some degree. Second, soil available nutrients are sensitive to soil moisture (He and Dijkstra 2014), and thus, even if we would have measured them, soil available N and P might be biased because we conducted sampling only during the hot summer.

### Climatic data

We obtained mean monthly temperature and precipitation data during the growing season (from May to October, the wettest half of the year) at a resolution of 30 arc‐seconds (~1 km^2^) from the WorldClim database (Hijmans et al. 2005).

The growing‐season wetness index (GWI) was used as a proxy for water availability. The GWI equals growing‐season precipitation (GP) divided by growing‐season potential evapotranspiration (GPE). Higher GWI values indicate lower aridity and higher water availability. GPE was calculated using the Thornthwaite equation (Thornthwaite and Mather 1955, Fang and Yoda 1990; see Appendix S1 for the detailed calculation process).

### Data analysis

**Figure 2:**
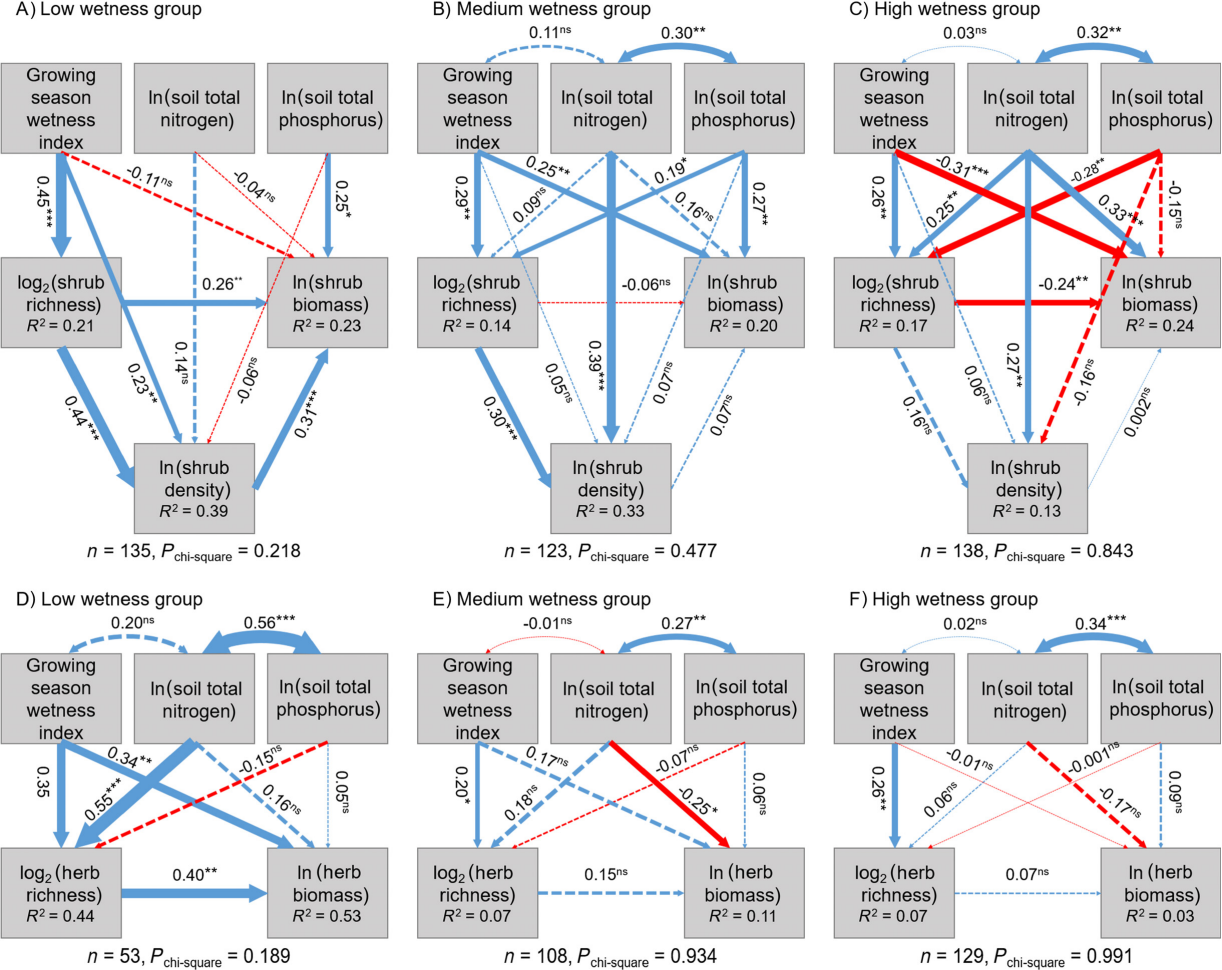
Structural equation models (SEMs) for total biomass of (A–C) shrubs and (D–F) herbs and three wetness conditions. Single‐headed arrows represent causal paths, and double‐headed arrows represent covariance paths. Standardized path coefficients and their significances (***P ≤ 0.001; **P ≤ 0.01; *P ≤ 0.05; ns*, P* > 0.05) are presented beside corresponding arrows, and arrow thickness is proportional to path coefficients (blue, positive; red, negative; solid, significant at *P* ≤ 0.05; dashed, not significant). The explained proportion of total variance (*R* ^2^) of each response variable is presented inside the respective box. Sample sizes and results of chi‐square tests are shown under each path diagram for each group.

To compare species‐richness–biomass relationships across the wetness gradient, we first divided all sites into three similar‐sized groups according to the GWI, with GWI < 0.63, 0.63 < GWI < 0.83, and GWI > 0.83 indicating low, medium, and high water availability, respectively. These thresholds were defined in such a way that the different environmental levels had comparable sample sizes. Second, structural equation models (SEMs) were fitted for the shrubs and herbs in each group (Fig. 2). Species richness and biomass were the response variables in these models. We log transformed these variables (species richness based on 2 and biomass based on *e*) so that the data would follow a normal distribution. The GWI and log‐transformed STN and STP (based on *e*) were used to represent the abiotic effects on species richness and biomass, while species richness was considered a biotic effect on biomass. We incorporated log‐transformed density (based on *e*) in the SEMs for shrubs. We assumed that density was affected by all abiotic factors as well as species richness and that biomass depended on the effect of density. We did not include density in the SEMs for herbs because herb density data were available for only one‐third of our sites due to the difficulty of identifying individuals. We also considered the covariance between the GWI and ln STN and between ln STN and ln STP because there were relatively strong correlations between them (correlation coefficients: GWI–ln STN, *r *=* *0.65; ln STN–ln STP, *r *=* *0.42; GWI–ln STP, *r *=* *0.17). The correlation matrices used to calculate the SEMs together with the scatter plots of the raw data are shown in Appendix S2: Fig. S1. There were 44 sites with no herbs at all, and we failed to obtain complete shrub or herb data at some sites, so 399 sites were used in the shrub models, and 290 sites were included in the herb models. Chi‐square tests were used to evaluate the results of the model fitting.

To disentangle the net effect of the biomass of the affecting life form and that of soil and climate on the species‐richness–biomass relationship of the responding life form, we first calculated the residuals of species richness and biomass after fitting them against abiotic factors (Eqs. (2) and (3)) to eliminate environmental effects (Freckleton 2002)

(2) lnShrub/Herb richness∼GWI+lnSTN+lnSTP

(3) lnShrub/Herb biomass∼GWI+lnSTN+lnSTP.

Then, we fitted the biomass residuals of the responding life form to the biomass residuals of the affecting life form, the species richness residuals of the responding life form and the interaction between them (Eqs. (4) and (5)):

(4) $$\begin{array}{cc} & \text{Shrub biomass residuals}∼\text{Herb biomass}\\ & \;\text{residuals}+\text{Shrub richness residuals}+\text{Herb}\\ & \;\text{biomass residuals}:\text{Shrub richness residuals} \end{array}$$

(5) $$\begin{array}{cc} & \text{Herb biomass residuals}∼\text{Shrub biomass}\\ & \text{residuals}+\text{Herb}\;\text{richness residuals}+\text{Shrub}\\ & \text{biomass residuals}:\text{Herb richness residuals}. \end{array}$$

After excluding sites with missing data, 281 sites remained for use in these two models. When applying the *F* test, we corrected for the degrees of freedom by subtracting the degrees of freedom already fitted in the previous models (Eqs. (2) and (3)), so the total degrees of freedom in the models were 277 instead of 280.

Significant interaction terms indicate significant influences of the affecting life form's biomass on the species‐richness–biomass relationship of the responding life form after excluding the soil and climatic effects. We then compared the species‐richness–biomass relationships of each life form at different biomass residual levels of the other life form. We divided all sites into three similar‐sized groups according to the herb biomass residuals or shrub biomass residuals.

Afterward, we performed all of the above analyses again with aboveground biomass and belowground biomass to detect how the above‐ and belowground processes affected the species‐richness–biomass relationships.

All analyses were conducted using R 3.4.0 (R Core Team 2017) and the SEM analyses were conducted with the lavaan package (Rosseel 2012).

## Results

### Species‐richness–biomass relationships under different wetness conditions

All initial path models passed the chi‐square test (*P* > 0.1, Fig. 2), except for that of shrubs under the driest conditions. Thus, we dropped the covariance links among environmental variables and nonsignificant causal links between soil nutrients and shrub richness from the initial model to reach *P* > 0.1 for the shrub model under the driest conditions. The remaining causal links in the updated model remained consistent with those in the initial model (Fig. 2A; Appendix S2: Fig. S2). Although environmental effects on richness, density and biomass varied across the wetness gradient, the GWI had a consistently positive effect on plant richness (Fig. 2). Shrub richness had a positive effect on shrub density under low or medium water availability, but shrub density positively affected the shrub biomass only under a low water supply. The direct effect of shrub richness on shrub biomass changed from positive to negative when the moisture increased (Fig. 2A–C), while the effect of herb richness on herb biomass changed from positive to nonsignificant under those conditions (Fig. 2D–F).

The SEM results for above‐ and belowground biomass (Appendix S2: Figs. S3 and S4) were similar to the results for total biomass. However, the shrub density had a marginally negative effect on aboveground biomass under medium and high wetness (*P* = 0.06 and *P* = 0.05, respectively; Appendix S2: Fig. S3B, C). In addition, a noticeable negative effect of herb richness on the aboveground biomass was found under medium and high water availability (*P* < 0.05 and *P* = 0.07, respectively; Appendix S2: Fig. S3E, F), but the effect on belowground biomass remained nonsignificant across the wetness gradient (Appendix S2: Fig. S4D–F).

### Residual species‐richness–biomass relationships of one life form at different biomass levels of the other life form

**Figure 3:**
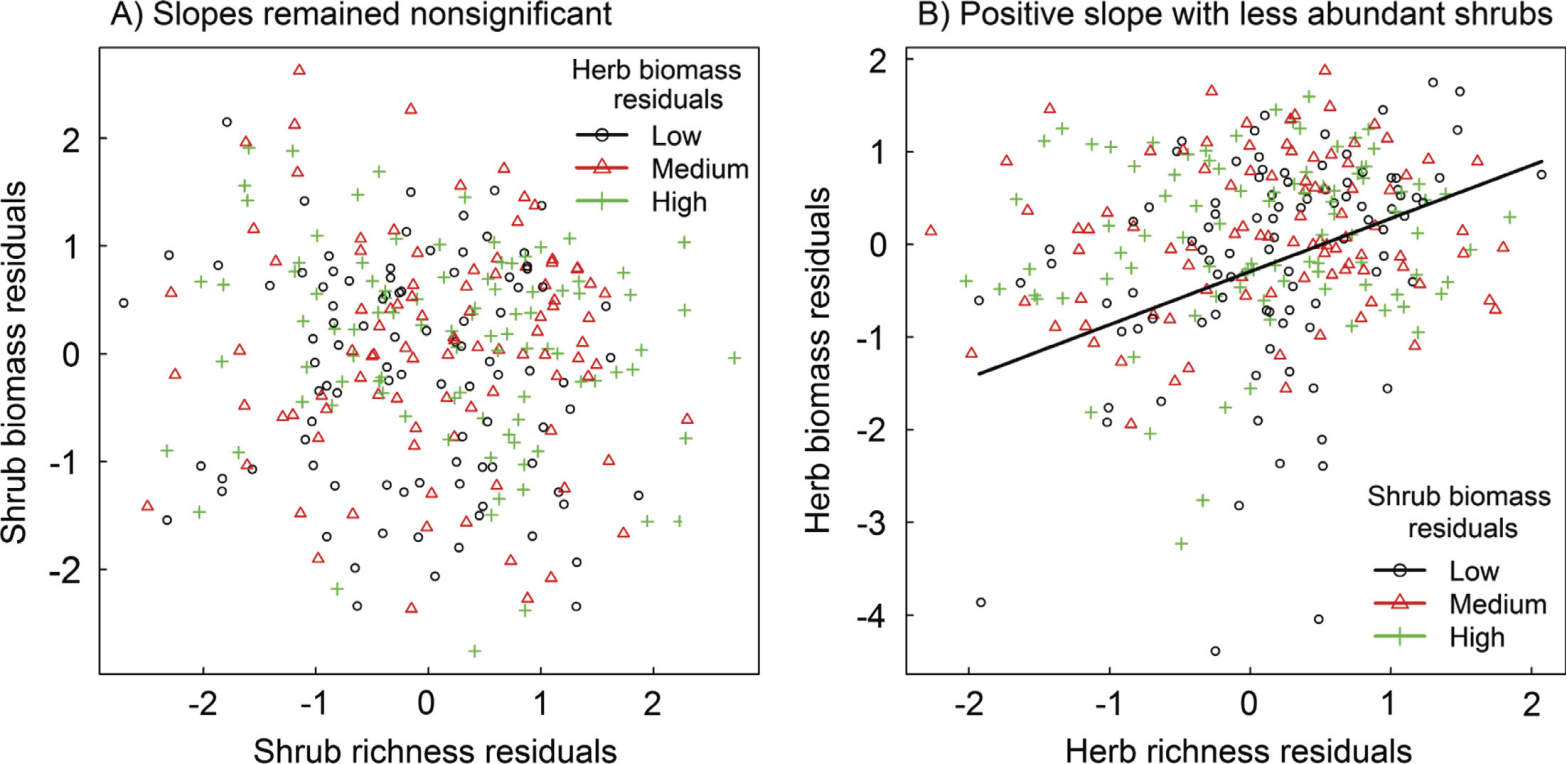
Residual species‐richness–biomass relationships of (A) shrubs and (B) herbs at different residual biomass levels of the other life form. Solid lines represent significant slopes (*P* ≤ 0.05); nonsignificant relationships (*P* > 0.05) are not shown.

The shrub biomass residuals (after correcting for abiotic variables; see Eqs. (2) and (3)) were positively related to the herb biomass residuals but not to the shrub richness residuals, and there was no interaction between the two (Table 1). In other words, the residual species‐richness–biomass relationship for shrubs was not significant after correcting for the abiotic variables (see Eqs. (2) and (3)), and the relationship itself was not significantly modified by competition from herbs. In contrast, the residual species‐richness–biomass relationship was significantly positive for herbs and significantly declined with increased shrub biomass residuals (Table 2, second and third row), which had a significant main effect on residual herb biomass (first row in Table 2). That is, shrub biomass was beneficial for herbs, suggesting facilitation by improving herb biomass, and reduced the dependency of herb biomass on herb species richness. In agreement with these residual models, the slopes of the species‐richness–biomass relationships of shrubs remained nonsignificant when the data were split into three equally sized groups according to the herb biomass residuals (Fig. 3A). However, when a similar split according to shrub biomass was made for the species‐richness–biomass relationship of herbs, it was significantly positive in the group with the lowest shrub biomass residuals, which represents the most stressful conditions for herbs (Fig. 3B).

**Table 1:** ANOVA parameters and regression coefficients in the modeling of shrub biomass residuals using Eq. (4)

Parameter	df	SS	*P* (*F* test)	Estimate
Herb biomass residuals	1	7.26	**<0.01**	0.16
Shrub richness residuals	1	1.91	0.16	−0.08
Interaction	1	0.20	0.64	0.03
Residuals	274	265.45		

*Notes:* df, degrees of freedom; SS, sum of squares. The number in boldface type denotes a significant relationship according to the *P* value.

**Table 2:** ANOVA parameters and regression coefficients in the modeling of herb biomass residuals using Eq. (5)

Parameter	df	SS	*P* (*F* test)	Estimate
Shrub biomass residuals	1	7.22	**<0.01**	0.17
Herb richness residuals	1	13.00	**<0.001**	0.27
Interaction	1	3.62	**0.05**	−0.14
Residuals	274	249.74		

*Notes:* For abbreviations, see Table 1. The numbers in boldface type denote significant relationships according to the *P* values.

When above‐ and belowground biomass were considered separately, we found that a negative change in the slope for herbs with increasing shrub biomass residuals existed for both above‐ and belowground biomass (Appendix S2: Tables S1–S4, Figs. S5 and S6).

## Discussion

Based on a large data set including the species composition and biomass of shrubland ecosystems, we compared the species‐richness–biomass relationships of two coexisting life forms and their responses to a water availability gradient. Increased water availability, i.e., release from abiotic stress, had a negative effect on the slope of the species‐richness–biomass relationships for both shrubs and herbs, supporting our first hypothesis in *Introduction*. Our second hypothesis was that a similar reduction of positive species‐richness–biomass relationships could occur when environmental stress is reduced due to facilitation. This hypothesis was also supported by our finding that, after correcting for abiotic variables, the residual species‐richness–biomass relationship for herbs was positive under the presumed stressful conditions with low shrub biomass and therefore low levels of facilitation than that under more beneficial conditions with medium or high shrub biomass. In the following section, we discuss these findings in more detail.

### Biodiversity–biomass relationships under different wetness conditions

In agreement with our first hypothesis, we found weaker species‐richness–biomass relationships with a greater water supply (Fig. 2). We also found that environmental effects on species richness and biomass could change with the moisture level, likely due to a change in the dominant limiting factors. For example, STP negatively affected the shrub richness, and the GWI negatively affected the shrub biomass under a higher moisture level. The former could be related to competitive exclusion (Hautier et al. 2009, Siddique et al. 2010) and the latter to decreased biomass production because of reduced light availability, poor soil drainage, or limited evapotranspiration (Schuur 2003, Nijp et al. 2015, Paquette et al. 2018).

Indeed, some studies have suggested a reverse causal link from productivity or biomass to biodiversity (Grace et al. 2007, 2016, Paquette and Messier 2011). We cannot exclude the possibility of this reverse causality in our study, but we can also still find some evidence that the causality was such that biodiversity affected biomass. First, some studies have suggested that the biodiversity of woody plants could be a driver of vegetation carbon storage (Ruiz‐Jaen and Potvin 2011, Wardle et al. 2012, Cavanaugh et al. 2014, Wu et al. 2015, Chen et al. 2018). Second, when shrub density was incorporated into the models, we had a clear view that shrub diversity promoted biomass partly by increasing plant density under low water availability (Fig. 2A), which concurs with previous experimental (Marquard et al. 2009) and comparative studies (Baruffol et al. 2013). These studies found that the effects of biodiversity on productivity occurred at least in part due to diversity‐induced increases in density. Biodiversity promotes plant density because individual plants in a more diverse community are less likely to grow with conspecific individuals, and their germination and establishment could be easier as a consequence owing to less niche overlap with their direct neighbors (Marquard et al. 2009). After accounting for density, the positive effect of shrub richness on biomass (Fig. 2A) implied that shrub richness can boost biomass through mechanisms other than increased density; for example, an increase in individual growth (Baruffol et al. 2013). However, accompanied by an increase in individual density, intensified competition could occur in terms of lower individual performance or a higher mortality rate (He et al. 2005, Marquard et al. 2009). The positive effects of richness on density and of density on biomass disappeared when water availability became higher (Fig. 2B, C), likely because density reached a plateau, and the effect of a smaller size of individuals offset the effect of increased density on biomass. If we only look at aboveground biomass, density had a marginally negative effect on biomass (Appendix S2: Fig. S3B, C, *P* = 0.06 under medium wetness and *P* = 0.05 under high wetness), indicating more limited shrub growth under increasing density, likely owing to increased shading and light competition.

Unfortunately, due to the lack of herb density data, we were unable to evaluate the role of density in the BEF relationships of herbs, but we still detected a decrease in the positive effect of biodiversity on biomass with increasing wetness (Fig. 2D–F). We also found that aboveground processes rather than belowground processes were likely responsible for this decrease (Appendix S2: Figs. S3D–F and S4D–F), suggesting the involvement of light competition.

The negative species‐richness–biomass relationships for shrubs (Fig. 2C) and herbs (Appendix S2: Fig. S3E, F) under better conditions indicate the occurrence of intensified competitive exclusion of subordinate shrub species (Bond and Chase 2002, Warren et al. 2009). One possible explanation for the observed negative effects is the relative importance of niche complementarity and competitive exclusion, as suggested in previous studies (Warren et al. 2009, Paquette and Messier 2011, Wu et al. 2015). It is conceivable that a reversal of causality or feedback from biomass to biodiversity could occur as environmental stress is alleviated (Grace et al. 2007, 2016), but the influence could be relatively weak because biodiversity is usually controlled by environmental factors and successional stages at large spatial scales (Mittelbach et al. 2001, Adair et al. 2018).

In addition to the previously mentioned observational studies (Paquette and Messier 2011, Potter and Woodall 2014, Wu et al. 2015), our interpretation is also supported by some experimental studies indicating reduced complementarity effects or weakened positive BEF relationships (Mulder et al. 2001, Zhang and Zhang 2006, García et al. 2018) under more favorable conditions, even though other experiments on herbaceous plants (He et al. 2002, Fridley 2003, Wacker et al. 2009, Yin et al. 2017) found the opposite. It is conceivable that the difference in results between some of these grassland experiments and our observational study is related to the fact that the experiments were conducted over a short time, which does not allow the detection of the effects of gradual changes in community composition following increased competitive interactions (Hautier et al. 2009, Bai et al. 2010). Another possible reason for the conflicting results may be that the design of manipulative experiments often reinforces the role of complementarity effects but weakens the role of competition in comparison with those that occur in natural ecosystems (Jiang et al. 2009); thus, the less positive BEF relationships driven by competitive exclusion are not well represented in these experiments.

### Biodiversity–biomass relationships of one life form at different levels of biomass of the other life form

In support of our second hypothesis, we found that changes in the biomass of the shrub layer did indeed affect the species‐richness–biomass relationship of the herbaceous community but not the other way around (see Fig. 3, Tables 1, 2). The nonsignificant residual species‐richness–biomass relationship of shrubs (second row in Table 1) could be attributed to that shrubs in the driest habitats, which showed a positive BEF relationship (Fig. 2A), were not considered due to the absence of coexisting herbs. The positive relationship between shrub biomass residuals and herb biomass residuals, for both above‐ and belowground biomass, indicates facilitation between these two coexisting life forms. The aboveground facilitation tended to be stronger than the belowground facilitation, as shown by the larger sum‐of‐squares proportion and regression coefficients (see Appendix S2: Tables S1–S4). Considering that shrubs were the dominant life form in the vegetation studied here and that results from previous studies also found facilitation of herbs by woody plants (Gómez‐Aparicio 2009, Cuesta et al. 2010, Dohn et al. 2013, Mazía et al. 2016), we suggest that, in our case, the facilitation occurred from shrubs to herbs. For example, aboveground facilitation could be related to more abundant shrubs providing more shade to retain soil water and more litter input for herbs (Maestre et al. 2003, Dohn et al. 2013), and factors such as increased soil microbial activity, higher concentrations of root exudates, or the occurrence of mycorrhizal networks might be involved in belowground facilitation by shrubs (Espeland and Rice 2007). The declining BEF slope for herbs with increasing shrub biomass might be associated with two aspects: improved living conditions could result in stronger competition among herbs, as we have discussed above, or herbaceous plants could be less dependent on biodiversity to achieve high biomass under increasing facilitation.

Previous studies on positive interactions between plants often found effects on the growth, survival, pollination, and other characteristics of beneficiaries (Maestre et al. 2001, Ghazoul 2006, Husheer et al. 2006, Kunstler et al. 2006, Cavieres and Badano 2009) but rarely considered the BEF relationships of “beneficiaries.” An exception is Badano and Marquet (2008), who found a more positive BEF relationship in the presence of facilitation from an alpine cushion plant. This difference from our own results is likely caused by the more severe environmental stress in alpine ecosystems, where competition between beneficiaries may still be limited (Grime 2001). In our study, the consequence of facilitation was likely stronger competitive exclusion among beneficiaries, so the fitness of some herbaceous species may still have been suppressed, even if facilitation from shrubs occurred (Schöb et al. 2013b).

**Figure 4:**
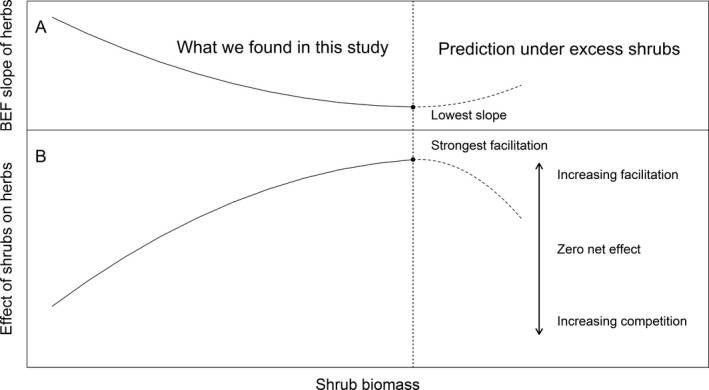
Diagram showing (A) the biodiversity–ecosystem‐functioning (BEF) slope of herbs and (B) the effect of shrubs on herbs along a gradient of shrub biomass. The vertical dashed line, which divides the diagram into two stages, indicates the value of shrub biomass at which shrubs have the strongest facilitative effects on herbs and the BEF slope of herbs is lowest. The left part of this diagram expresses our results, whereas the right part is what we expect to happen under an even higher shrub biomass than the maximum observed in the present study.

We conceptualize the change in the residual species‐richness–biomass relationship for herbs with increasing shrub biomass as a unimodal curve along the gradient of shrub biomass (Fig. 4). Given that the analysis accounted for the effects of the changing environmental conditions, the BEF slope of herbaceous plants decreased with increasing facilitation owing to the continuous improvement of living conditions caused by the higher abundance of shrubs (left part of Fig. 4). However, the facilitative effects on herbs may be reversed under even higher shrub biomass than that occurring in our study, potentially due to competition for water, nutrients, or light (Dohn et al. 2013, Noumi et al. 2016). Therefore, the facilitation of herbs by shrubs could reach a peak, and the BEF slope of herbs could again become positive due to increased environmental stress (right part of Fig. 4).

## Conclusions

We found that increasing water availability reduced positive biodiversity–biomass relationships for both shrubs and herbs, indicating stronger competition within life forms under more beneficial conditions. Furthermore, the positive species‐richness–biomass relationships for herbaceous plants decreased with increasing shrub facilitation. These findings show that stress resulting from the absence of an abiotic factor, water, and that resulting from the absence of a biotic factor, facilitation, can have the predicted effect on BEF relationships. Our research sheds light on the combined effects of biodiversity and the abiotic environmental context on biomass and thus carbon stock accumulation in shrublands. To maintain carbon stocks under scenarios of future drier climates due to global warming (Dai 2013), it will therefore be important to maintain plant diversity in these northern Chinese shrublands. Finally, our study confirms the possibility of using shrubs as nurse plants to alleviate environmental stress in the restoration of degraded habitats.
