# Supplementary material for: Increasing water availability and facilitation weaken biodiversity–biomass relationships in shrublands
Source: Ecology. 2019 Feb 25;100(3):e02624. doi: 10.1002/ecy.2624 (PMC6850503; doi:10.1002/ecy.2624)
Supplement: Supplementary file 1 [file ECY-100-na-s001.pdf]

Supporting Information. Guo, Y., C. Schöb, W. Ma, A. Mohammat, H. Liu, S. Yu, Y. Jiang, B. Schmid, and Z. Tang. 2019. Increasing water availability and facilitation weaken biodiversity–biomass relationships in shrublands. *Ecology.*

Appendix S1. Calculation of growing season evapotranspiration (GPE).

First, for each month from May to October, the calculation of unjustified potential evapotranspiration (*uPE*) differed according to mean monthly temperature (*t*):

$$I = \sum_{n=1}^{12} \left(\frac{t}{5}\right)^{1.514} \quad \text{(Eq. S1)}$$

$$a = (0.675 \times I^3 - 77.1 \times I^2 + 17920 \times I + 492390) \times 10^{-6} \quad \text{(Eq. S2)}$$

$$\begin{cases} uPE = 188 \times \left(1 - e^{-0.0000061 \times t^{3.734}}\right), t > 26.5 \\ uPE = 16 \times (10 \times t/I)^a, 0 < t < 26.5 \\ uPE = 0, t \le 0 \end{cases} \quad \text{(Eq. S3)}$$

Second, we calculated adjusted potential evapotranspiration (*PE*) based on *uPE* and site latitude (*L*):

$$PE_{\text{May}} = uPE \times (-0.0098 \times L + 0.8609)^{-\frac{1}{3.5}} \quad \text{(Eq. S4)}$$

$$PE_{\text{Jun}} = uPE \times (-0.0101 \times L + 0.9739)^{-\frac{1}{2.5}} \quad \text{(Eq. S5)}$$

$$PE_{\text{Jul}} = uPE \times (-0.0088 \times L + 0.9024)^{-\frac{1}{2.5}} \quad \text{(Eq. S6)}$$

$$PE_{\text{Aug}} = uPE \times (-0.0089 \times L + 0.8481)^{-\frac{1}{4.2}} \quad \text{(Eq. S7)}$$

$$PE_{\text{Sep}} = uPE \times (-0.0104 \times L + 0.5984)^{-\frac{1}{48}} \quad \text{(Eq. S8)}$$

$$PE_{\text{Oct}} = uPE \times (-0.016 \times L + 1.3396)^{\frac{1}{8}} \quad \text{(Eq. S9)}$$

Finally, GPE is the sum of the *PE*s of these six months.
