# Supplementary material for: Increasing water availability and facilitation weaken biodiversity–biomass relationships in shrublands
Source: Ecology. 2019 Feb 25;100(3):e02624. doi: 10.1002/ecy.2624 (PMC6850503; doi:10.1002/ecy.2624)

Supporting Information. Guo, Y., C. Schöb, W. Ma, A. Mohammat, H. Liu, S. Yu, Y. Jiang, B. Schmid, and Z. Tang. 2019. Increasing water availability and facilitation weaken biodiversity–biomass relationships in shrublands. *Ecology.*

Appendix S2. Supplementary tables and figures

Table S1. ANOVA parameters and regression coefficients in the modeling of shrub aboveground biomass residuals using Eq. 4

	DF	SS	P (F-test)	Estimate
Herb aboveground biomass residuals	1	46.15	**<0.001**	0.34
Shrub richness residuals	1	0.04	0.83	<0.01
Interaction	1	1.81	0.15	0.07
Residuals	302	266.87		

Abbreviations: DF, degrees of freedom; SS, sum of squares. The number in bold denotes a significant relationship according to the P value.

Table S2. ANOVA parameters and regression coefficients in the modeling of herb aboveground biomass residuals using Eq. 5

	DF	SS	P (F-test)	Estimate
Shrub aboveground biomass residuals	1	55.01	**<0.001**	0.42
Herb richness residuals	1	0.75	0.39	0.06
Interaction	1	11.94	**<0.001**	-0.23
Residuals	297	295.48		

Abbreviations: see Table S1. The numbers in bold denote significant relationships according to the P values.

Table S3. ANOVA parameters and regression coefficients in the modeling of shrub belowground biomass residuals using Eq. 4

	DF	SS	P (F-test)	Estimate
Herb belowground biomass residuals	1	4.76	**0.05**	0.12
Shrub richness residuals	1	4.08	0.07	-0.11
Interaction	1	0.52	0.51	-0.03
Residuals	293	349.18		

Abbreviations: see Table S1. The number in bold denotes a significant relationship according to the P value.

Table S4. ANOVA parameters and regression coefficients in the modeling of herb belowground biomass residuals using Eq. 5

	DF	SS	P (F-test)	Estimate
Shrub belowground biomass residuals	1	5.35	**0.04**	0.11
Herb richness residuals	1	21.99	**<0.001**	0.36
Interaction	1	11.00	**<0.01**	-0.21
Residuals	293	364.54		

Abbreviations: see Table S1. The numbers in bold denote significant relationships according to the P values.

Supplementary figures

Fig. S1. Correlation matrices between growing-season wetness index (GWI), soil total nitrogen (STN), soil total phosphorus (STP), shrub biomass (SB), shrub richness (SR), shrub density (SD), herb biomass (HB) and herb richness (HR) for the low wetness group (A), the medium wetness group (B) and the high wetness group (C). The values of correlation coefficients are shown in the upper triangles. All variables except the GWI were log-transformed.

Fig. S1

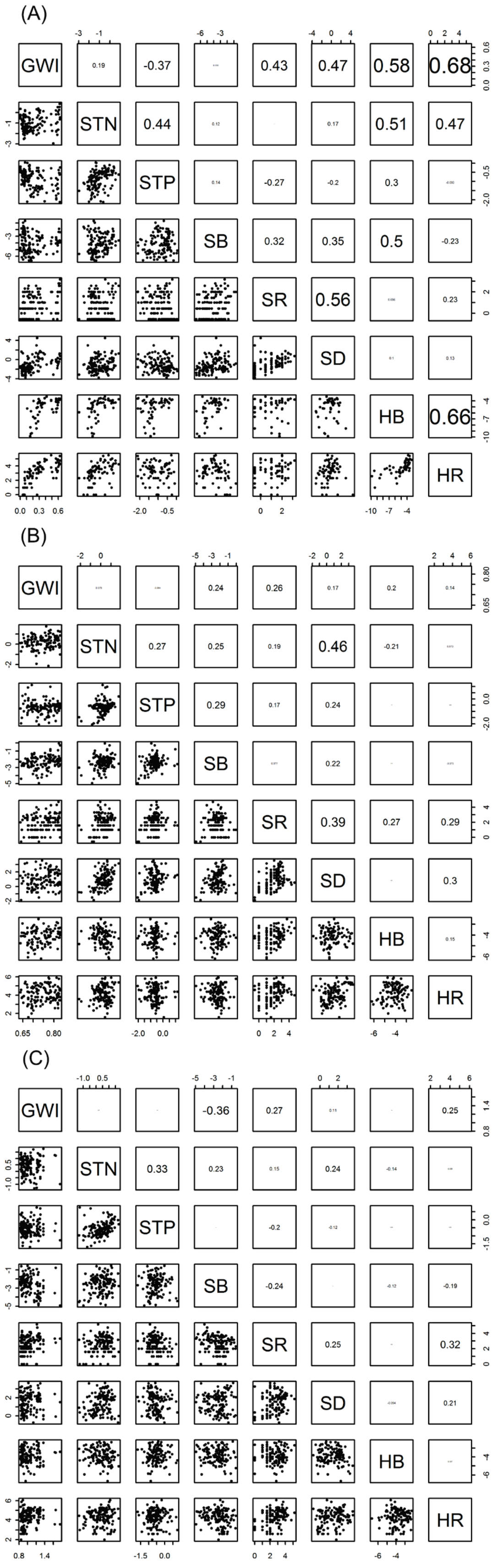

(A)
GWI 0.19 -0.37 0.43 0.47 0.58 0.68
STN 0.44 0.12 0.17 0.51 0.47
STP 0.14 -0.27 -0.2 0.3
SB 0.32 0.35 0.5 -0.23
SR 0.56 0.23
SD 0.1 0.13
HB 0.66
HR
(B)
GWI 0.24 0.26 0.17 0.2 0.14
STN 0.27 0.25 0.19 0.46 -0.21
STP 0.29 0.17 0.24
SB 0.22
SR 0.39 0.27 0.29
SD 0.3
HB 0.15
HR
(C)
GWI -0.36 0.27 0.25
STN 0.33 0.23 0.15 0.24 -0.14
STP -0.2 -0.12
SB -0.24 -0.12 -0.19
SR 0.25 0.32
SD 0.21
HB
HR

Fig. S2. Structural equation model (SEM) for total biomass of shrubs under low wetness conditions. Single-headed arrows represent causal paths and double-headed arrows represent covariance paths. Standardized path coefficients and their significances ($^{***}P \leq 0.001$; $^{**}P \leq 0.01$; $^{*}P \leq 0.05$; ns, $P > 0.05$) are presented beside corresponding arrows, and arrow thickness is proportional to path coefficients (blue, positive; red, negative; solid, significant at $P \leq 0.05$; dashed, not significant). The explained proportion of total variance (R^2) of each response variable is presented inside the respective box. Sample size and result of chi-square test are shown under the path diagram.

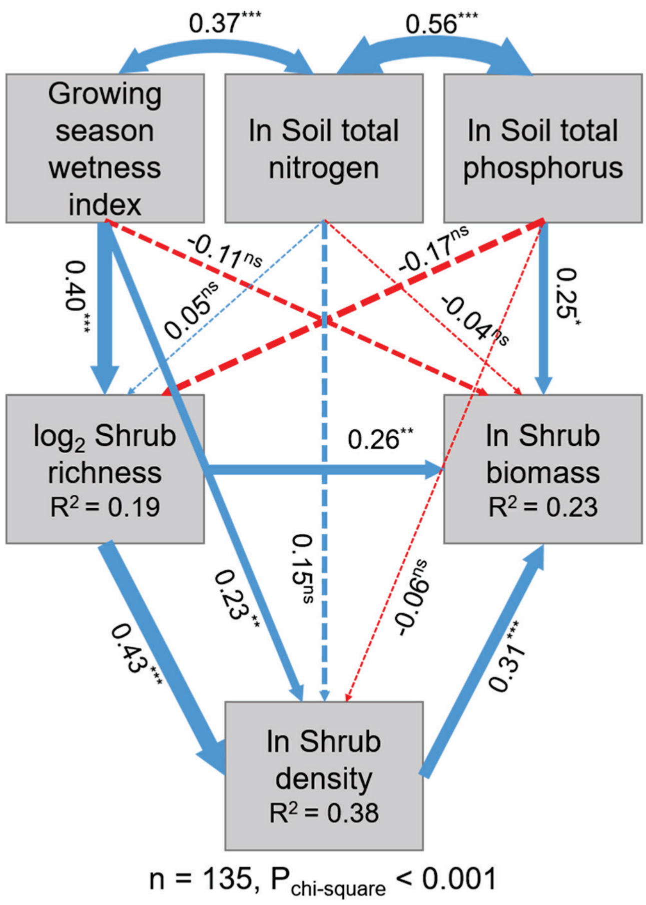

Fig. S3. Structural equation models (SEMs) for aboveground biomass of shrubs (A–C) and herbs (D–F) and three wetness conditions. Single-headed arrows represent causal paths, and double-headed arrows represent covariance paths. Standardized path coefficients and their significances ($^{***}P \leq 0.001$; $^{**}P \leq 0.01$; $^{*}P \leq 0.05$; ns, $P > 0.05$) are presented beside corresponding arrows, and arrow thickness is proportional to path coefficients (blue, positive; red, negative; solid, significant at $P \leq 0.05$; dashed, not significant). The explained proportion of total variance (R^2) of each response variable is presented inside the respective box. Sample sizes and results of chi-square tests are shown under each path diagram for each group.

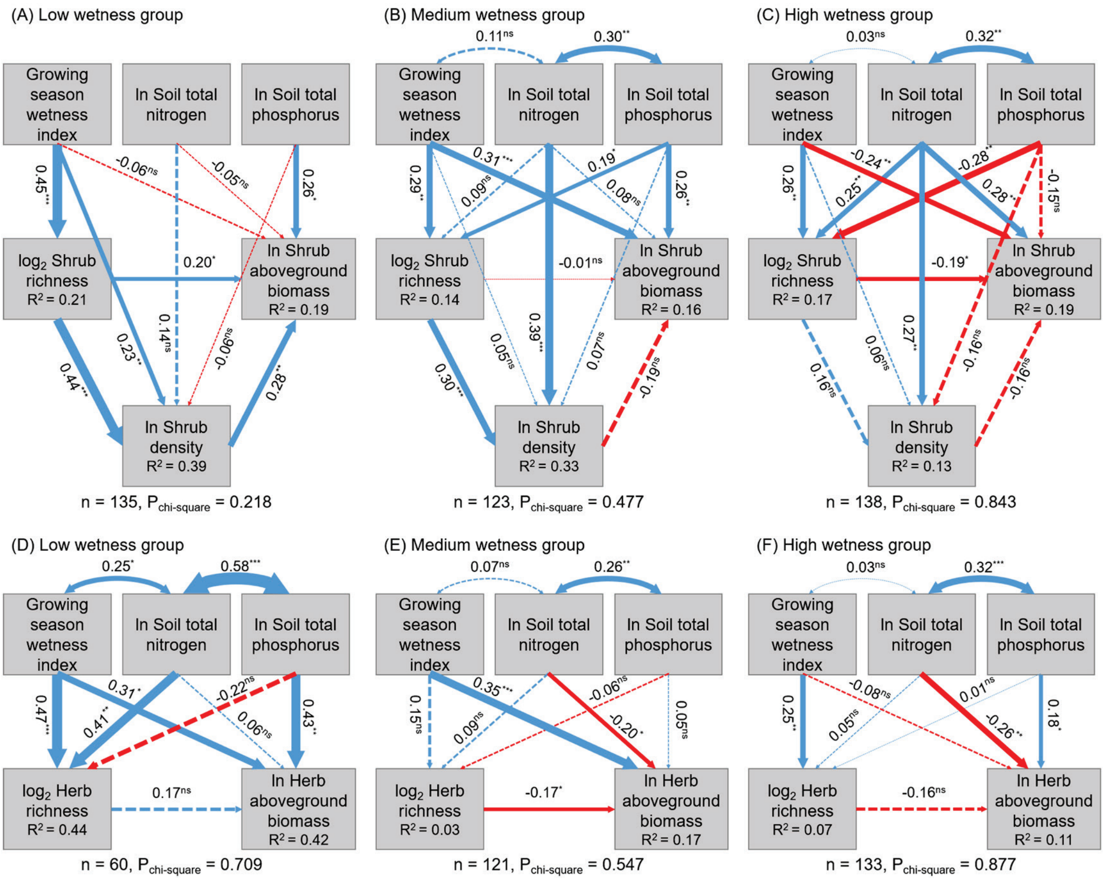

Fig. S4. Structural equation models (SEMs) for belowground biomass of shrubs (A–C) and herbs (D–F) and three wetness conditions. Single-headed arrows represent causal paths, and double-headed arrows represent covariance paths. Standardized path coefficients and their significances ($^{***}P \leq 0.001$; $^{**}P \leq 0.01$; $^{*}P \leq 0.05$; ns, $P > 0.05$) are presented beside corresponding arrows, and arrow thickness is proportional to path coefficients (blue, positive; red, negative; solid, significant at $P \leq 0.05$; dashed, not significant). The explained proportion of total variance (R^2) of each response variable is presented inside the respective box. Sample sizes and results of chi-square tests are shown under each path diagram for each group.

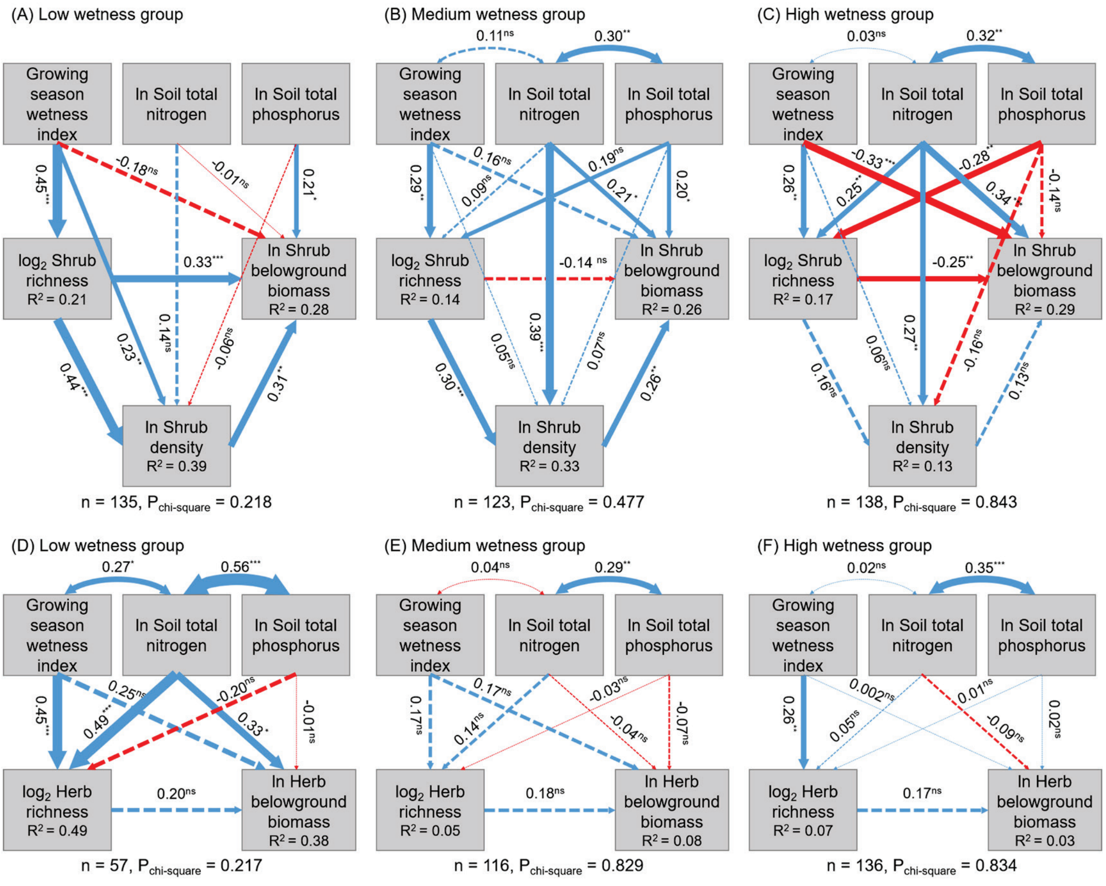

Fig. S5. Residual species-richness aboveground biomass relationships of shrubs (A) and herbs (B) at different residual biomass levels of the other life form. Solid lines represent significant slopes (P ≤ 0.05); nonsignificant relationships (P > 0.05) are not shown.

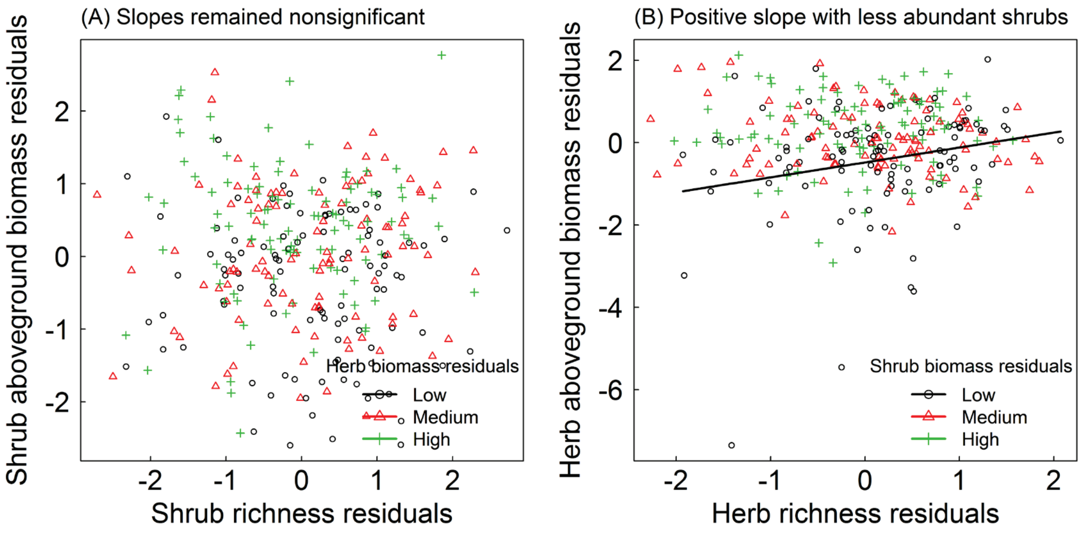

Fig. S6. Residual species-richness belowground biomass relationships of shrubs (A) and herbs (B) at different residual biomass levels of the other life form. Solid lines represent significant slopes ($P \leq 0.05$); nonsignificant relationships ($P > 0.05$) are not shown.

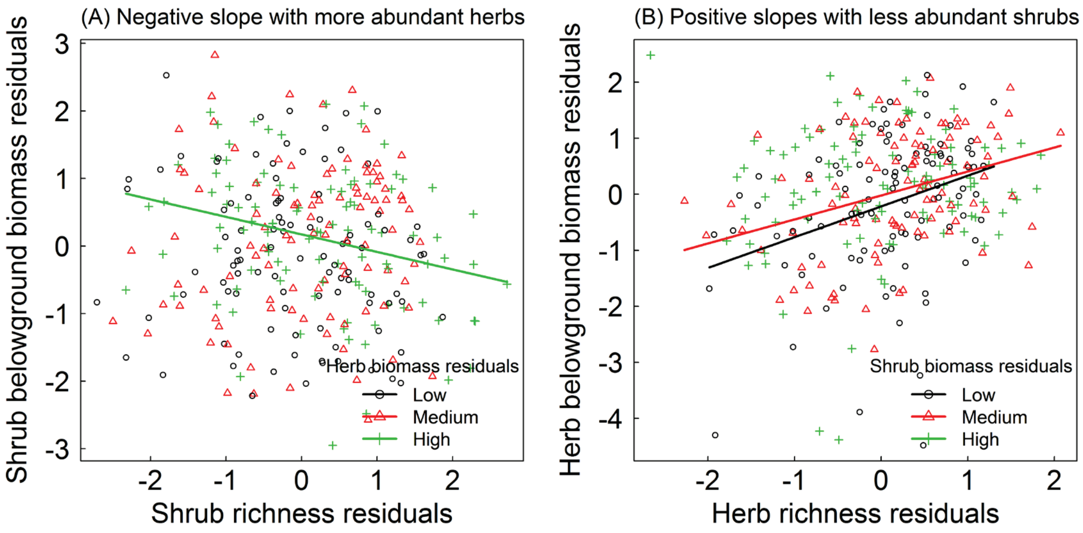

Supplement: Supplementary file 2 [file ECY-100-na-s002.pdf]
