# Supplementary material for: Increasing water availability and facilitation weaken biodiversity–biomass relationships in shrublands
Source: Ecology. 2019 Feb 25;100(3):e02624. doi: 10.1002/ecy.2624 (PMC6850503; doi:10.1002/ecy.2624)
Supplement: Supplementary file 3 [file ECY-100-na-s003.pdf]

Metadata S1. Guo, Y., C. Schöb, W. Ma, A. Mohammat, H. Liu, S. Yu, Y. Jiang, B. Schmid, and Z. Tang. 2019. Increasing water availability and facilitation weaken biodiversity–biomass relationships in shrublands. *Ecology.*

Data S1

Supplementary dataset and R code for statistical analysis.

Authors

Yanpei Guo
Institute of Ecology, College of Urban and Environmental Sciences and Key Laboratory for Earth Surface Processes, Peking University, Beijing, China
guoyanpei@pku.edu.cn

Christian Schöb
Department of Environmental Systems Science, Swiss Federal Institute of Technology, ETH Zurich, Zurich, Switzerland
christian.schoeb@usys.ethz.ch

Wenhong Ma
School of Life Sciences, Inner Mongolia University, Hohhot, China
whmapku@126.com

Anwar Mohammat
Xinjiang Institute of Ecology and Geography, Chinese Academy of Sciences, Urumqi, China
anwar@ms.xjb.ac.cn

Hongyan Liu
Institute of Ecology, College of Urban and Environmental Sciences and Key Laboratory for Earth Surface Processes, Peking University, Beijing, China
lhy@urban.pku.edu.cn

Shunli Yu
State Key Laboratory of Vegetation and Environmental Changes, Institute of Botany, Chinese Academy of Sciences, Beijing, China
shunliyu@ibcas.ac.cn

Youxu Jiang
Key Laboratory of Forest Ecology and Environment, State Forestry Administration, Research Institute of Forest Ecology, Environment, and Protection, Chinese Academy of Forestry, Beijing, China
Jiangyx@caf.ac.cn

Bernhard Schmid
Department of Geography, University of Zurich, Zurich, Switzerland
bernhard.schmid@ieu.uzh.ch

Zhiyao Tang
Institute of Ecology, College of Urban and Environmental Sciences and Key Laboratory for Earth Surface Processes, Peking University, Beijing, China
zytang@urban.pku.edu.cn

File list (files found within DataS1.rar)

`DataS1.csv`
`code for analysis.R`

Description

`DataS1.csv` – List of shrubland sites with information about habitat conditions and communities.

site	Code name of each shrubland site
plotarea	Area of each plot (m^2)
WL.3	Three water availability levels according to GWI
MGT	Mean growing-season temperature (°C)
GPE	Growing-season potential evapotranspiration (mm)
GP	Growing-season precipitation (mm)
GWI	Growing-season wetness index
STN	Soil total nitrogen concentration (mg g^{-1})
STP	Soil total phosphorus concentration (mg g^{-1})
richness.h	Species richness of herbaceous plants
richness.so	Original species richness of shrubs
richness.s	Species richness of shrubs after correction for plot sizes
herbL	Aboveground biomass of herbaceous plants (Mg hm^{-2})
herbR	Belowground biomass of herbaceous plants (Mg hm^{-2})
hbiomass	Total biomass of herbaceous plants (Mg hm^{-2})
shrubR	Belowground biomass of shrubs (Mg hm^{-2})
sabove	Aboveground biomass of shrubs (Mg hm^{-2})
sbiomass	Total biomass of shrubs (Mg hm^{-2})
sID	Individual density of shrubs (m^{-2})

`code for analysis.R` - R script for structural equation modelling and residual regression. Annotations of code are included throughout the script.
